# Supplementary material for: Digital Biomarkers for Personalized Nutrition: Predicting Meal Moments and Interstitial Glucose with Non-Invasive, Wearable Technologies
Source: Nutrients. 2022 Oct 24;14(21):4465. doi: 10.3390/nu14214465 (PMC9654068; doi:10.3390/nu14214465)
Supplement: Supplementary file 1 [file nutrients-14-04465-s001.zip › nutrients-1932182-supplementary.pdf]

Supplementary Table S1

Table S1. Feature importance of the different contextual modalities, activity, nutrition, and sleep, as well as the between-individual variation.

Feature	Weight
**ee_last_24h**	0.101717
**acn_last_8h**	0.084459
**similarity_to_tno14**	0.080656
**ahr_last_24h**	0.067866
**acn_last_3h**	0.065052
**ahr_last_8h**	0.062497
**calories_last_3h**	0.059207
**subject_11 ***	0.056915
**subject_15 ***	0.054008
**deep_sleep**	0.053423
**sleep_duration**	0.053098
**calories_from_carbohydrate_last_3h**	0.050084
**subject_22 ***	0.049044
**calories_last_8h**	0.047338
**acn_last_24h**	0.040274
**calories_from_carbohydrate_last_24h**	0.039837
**ee_last_8h**	0.034526

ee: energy expenditure, acn: acceleration, ahr: average heart rate. * Subject number was included as binary feature to account for unexplained subject-specific variance.
